# NRF2 Regulates Viability, Proliferation, Resistance to Oxidative Stress, and Differentiation of Murine Myoblasts and Muscle Satellite Cells

**DOI:** 10.3390/cells11203321

**Published:** 2022-10-21

**Authors:** Iwona Bronisz-Budzyńska, Magdalena Kozakowska, Katarzyna Pietraszek-Gremplewicz, Magdalena Madej, Alicja Józkowicz, Agnieszka Łoboda, Józef Dulak

**Affiliations:** Department of Medical Biotechnology, Faculty of Biochemistry, Biophysics and Biotechnology, Jagiellonian University, 30-387 Kraków, Poland

**Keywords:** muscle regeneration, myoblasts, NRF2, oxidative stress, satellite cells

## Abstract

Increased oxidative stress can slow down the regeneration of skeletal muscle and affect the activity of muscle satellite cells (mSCs). Therefore, we evaluated the role of the NRF2 transcription factor (encoded by the *Nfe2l2* gene), the main regulator of the antioxidant response, in muscle cell biology. We used (i) an immortalized murine myoblast cell line (C2C12) with stable overexpression of NRF2 and (ii) primary mSCs isolated from wild-type and *Nfe2l2* (transcriptionally)-deficient mice (*Nfe2l2*^tKO^). NRF2 promoted myoblast proliferation and viability under oxidative stress conditions and decreased the production of reactive oxygen species. Furthermore, NRF2 overexpression inhibited C2C12 cell differentiation by down-regulating the expression of myogenic regulatory factors (MRFs) and muscle-specific microRNAs. We also showed that NRF2 is indispensable for the viability of mSCs since the lack of its transcriptional activity caused high mortality of cells cultured in vitro under normoxic conditions. Concomitantly, *Nfe2l2*^tKO^ mSCs grown and differentiated under hypoxic conditions were viable and much more differentiated compared to cells isolated from wild-type mice. Taken together, NRF2 significantly influences the properties of myoblasts and muscle satellite cells. This effect might be modulated by the muscle microenvironment.

## 1. Introduction

Nuclear factor erythroid 2 (NF-E2)-related factor 2 (NRF2; encoded by *Nfe2l2* gene) belongs to the Cap‘n’collar (CNC)-basic leucine zipper (bZIP) family of transcription factors and regulates the expression of numerous genes involved in oxidative stress response, lipid metabolism, detoxication, gene transcription, and metabolism of heme and iron [1]. This regulation is mediated by binding to the antioxidant response element (ARE) sequence present in the promoter region of more than 250 genes [2]. Therefore, NRF2 is considered a crucial protein that protects cells from disturbed redox balance [3].

Elevated oxidative stress has significant molecular, structural, and functional implications for skeletal muscles. If exaggerated, the increase in the burst of reactive oxygen species (ROS) causes cell dysfunction and contributes to the progression of muscle diseases. For example, oxidative damage to skeletal muscle fibers can worsen the pathology of Duchenne muscular dystrophy (DMD), a disease caused by mutations in the structural protein, dystrophin [4,5].

In muscles, ROS can be involved in the activation of muscle satellite cells (mSCs), a bona fide population of adult stem cells indispensable for muscle repair, remodeling, and growth [6]. Although mSCs are mitotically quiescent in mature skeletal muscle, they undergo mitosis after trauma [7,8]. Furthermore, the myogenesis and activity of mSCs can be regulated by the hypoxic environment [9]. In response to injury, mSCs are activated, enter the cell cycle, and proliferate. This is accompanied by the migration of mSCs to damaged sites where they differentiate into myoblasts and fuse with destroyed fibers or with each other to form multinucleated myofibers de novo [10]. Newly formed myofibers are characterized by the expression of unique developmental myosin isoforms, such as embryonic myosin heavy chain (eMHC), encoded by myosin heavy chain 3 (*Myh3*), and have centrally localized nuclei, which is the main feature of regenerating myofibers [11]. The physiology and myogenic potential of mSCs are strictly controlled by paired box 7 (PAX7) transcriptional factor and myogenic regulatory factors (MRFs), including myogenic factor 5 (MYF5), myogenic determination factor (MYOD), myogenin, and muscle regulatory factor 4 (MRF4, also known as an MYF6—myogenic factor-6) [12]. In addition to MRFs, skeletal muscle myogenesis is regulated by microRNAs (miRNAs, miRs), especially a group of myomiRs (miR-1, miR133a/b, miR-206). Their transient expression during myoblast differentiation in vitro [13,14] and in vivo [15,16] negatively regulates the expression of target genes and is necessary for the normal development of muscle tissue.

The redox state influences the functions of mSCs. Therefore, their protection from oxidative damage is essential to ensure adequate muscle regeneration. It should be noted that initial attempts have already been made to define the role of NRF2 and NRF2-related genes in mSCs and skeletal muscles. Intriguingly, in vitro experiments revealed that in primary myoblasts, NRF2 had an anti-myogenic effect by decreasing *Myod1* and *Myog* mRNA expression, as well as anti-proliferative potential through down-regulating cell cycle-related genes and slowing down the rate of replication [17]. On the other hand, an increase in ROS production was evident along with antioxidant deregulation and oxidative stress-induced apoptosis in the skeletal muscle of aged NRF2-deficient mice [18,19]. Shelar et al. also showed impaired regeneration in NRF2-null animals after an acute injury caused by the injection of cardiotoxin (CTX) [20]. Furthermore, muscle regeneration was impaired in mice lacking transcriptionally active NRF2 after ischemia-reperfusion injury [21]. In our hands, the lack of NRF2 transcriptional activity has no profound impact on muscle pathology in both acute injury (CTX injection) and chronic disease (a mouse model of DMD) [22]; however, we have demonstrated the strong effect of heme oxygenase-1 (HO-1)—a heme-degrading enzyme that is regulated by NRF2 among others—on myoblast proliferation and differentiation and DMD progression [23,24].

In general, although some data indicate that the NRF2 system can regulate mSC proliferation and myogenesis, others suggest that it is dispensable or there are redundant genes or complementary pathways, that compensate for its loss in muscle and/or mSCs [22,25]. To shed more light on this aspect, we investigated the influence of NRF2 on muscle regeneration. Our study reveals that NRF2 affects the viability, proliferation, and differentiation of the murine myoblast C2C12 cell line and primary mSCs.

## 2. Materials and Methods

### 2.1. Generation of the C2C12 Cell Line with Stable Overexpression of NRF2

The C2C12 cell line, which is a subclone of C3H cells derived from mouse limb muscle, a model of activated satellite cells, was originally purchased from ATTC (CRL-1772). To generate C2C12 with NRF2 overexpression (C2C12-NRF2), the cells expressing the firefly luciferase gene (necessary for the in vivo experiments not described in this manuscript) were modified with the retroviral vector harboring a mouse NRF2 cDNA under the control of the CMV promoter and purified by selection on 0.8 µg/mL geneticin (G418; CytoGen GmbH, Wetzlar, Germany). The plasmid-containing mouse NRF2 cDNA was kindly gifted by Dr. Ken Itoh and Dr. Masayuki Yamamoto (University of Tsukuba, Tsukuba, Ibaraki, Japan) [26].

For a routine culture of C2C12 and C2C12-NRF2 cells, Dulbecco’s modified Eagle’s medium supplemented with glucose (25 mmol/L) (DMEM HG), 10% fetal bovine serum (FBS), penicillin (100 U/mL), and streptomycin (100 µg/mL) was used (GrM—growth medium). Cells were cultured under standard conditions (5% CO_2_, 37 °C, 95% humidity) and passaged every 2 to 4 days with 0.25% trypsin-EDTA (Gibco Waltham, MA, USA) to avoid full confluence.

### 2.2. Differentiation of C2C12 Cells

To induce differentiation, 100,000 cells/24 well plate or 200,000 cells/12 well plate were cultured for 24 h in GrM. The medium was then changed to differentiation medium (DM) containing DMEM HG, 2% horse serum (HS, PAA Laboratories, Cölbe, Germany), penicillin (100 U/mL), and streptomycin (100 µg/mL). After 5 days of culture, the level of cell differentiation was analyzed by immunofluorescent staining.

### 2.3. Isolation, Culture, and Differentiation of mSCs

To isolate mSCs by fluorescence-activated cell sorting (FACS), skeletal muscles from wild-type and *Nfe2l*2^tKO^ mice were prepared as previously described [24,27]. Mice with disrupted *Nfe2l2* gene on a C57Bl/6J background (originally developed by Prof. Masayuki Yamamoto [28] and further demonstrated by us to be a transcriptional knockout (*Nfe2l2*^tKO^) [29]), were bred from mice kindly provided by Prof. Antonio Cuadrado, in the animal facility of the Faculty of Biochemistry, Biophysics and Biotechnology, Jagiellonian University [30].

After staining with rat anti-mouse CD31-PE (MEC 13.3, eBioscience, San Diego, CA, USA), rat anti-mouse CD45-PE (30-F11, eBioscience), rat anti-mouse Sca1-PE-Cy7 (D7, eBioscience), and rat anti-mouse α7-integrin-APC (FAB3518A, R&D System, Minneapolis, MN, USA), mSCs were sorted by phenotype: CD45^−^CD31^−^Sca1^−^α7i^+^ (Supplementary Figure S1A). After sorting, cells were suspended in mSC growth medium (mSC GrM): DMEM HG, 20% FBS, 10% HS, penicillin (100 U/mL), streptomycin (100 µg/mL), and 5 ng/mL basic fibroblast growth factor (bFGF, PeproTech). Cells were seeded on a 96 well plate previously coated with gelatin (Sigma-Aldrich, St. Louis, MO, USA).

To induce differentiation, 10,000 cells were seeded in a 96 well plate in mSC GrM, and 24 h after isolation, it was replaced with mSC DM containing DMEM HG, penicillin/streptomycin, and 10% HS, and the cells were allowed to grow for 3 days (Supplementary Figure S1B).

To analyze mSC differentiation under hypoxic conditions, sorted cells were immediately seeded in a 96 well plate placed in a hypoxia chamber (0.5% O_2_, COY Laboratory, Grass Lake, MI, USA). The medium change and staining were also performed in the hypoxia chamber.

### 2.4. Cell Viability

C2C12 and C2C12-NRF2 cells were cultured in a 12 well plate and stimulated overnight with 0.5 mM and 1 mM H_2_O_2_. The next day, apoptotic and necrotic cells were stained using a commercially available kit containing propidium iodide (PI) and annexin V (TACS^TM^ Annexin V-FITC Apoptosis Detection Kit, R&D Systems) using the protocol provided by the manufacturer. The samples were acquired with a Fortessa flow cytometer and analyzed using FACSDiva software (BD Biosciences, San Diego, CA, USA).

### 2.5. Cell Proliferation

C2C12 and C2C12-NRF2 cells growing in a 12 well plate were fixed and permeabilized using BD IntraSure^TM^ Kit reagents (BD Biosciences). Intracellular staining was performed by adding rat anti-mouse Ki-67-FITC (eBioscience) diluted in PBS + 0.5% Triton100X. After co-staining with Hoechst (1:1000), cells were analyzed on a Fortessa flow cytometer using FACSDiva software (BD Biosciences). Gates were set on the appropriate fluorescent minus one (FMO) controls. Additionally, to evaluate cell proliferation, the incorporation of 5-bromo-2′-deoxyuridine (BrdU) was examined, based on the manufacturer’s instructions (Cell Proliferation ELISA, BrdU, Roche, Basel, Switzerland).

### 2.6. Production of Reactive Oxygen Species

C2C12 and C2C12-NRF2 cells were seeded at a density of 50,000 cells per well in a 12 well plate and analyzed the next day. Cells were prepared and stained with CellROX Green Reagent (Life Technologies, Carlsbad, CA, USA) according to the vendor’s instructions. Data were acquired with a Fortessa flow cytometer and analyzed using FACSDiva software (BD Biosciences).

### 2.7. Gene Expression Analysis

To isolate RNA, a modified Chomczynski–Sacchi method [31] with cell lysis and Fenozol reagent (A&A Biotechnology, Gdańsk, Poland) was used, followed by chloroform extraction and isopropanol precipitation. The concentration and quality of isolated RNA were determined spectrophotometrically using NanoDrop N-1000 (Thermo Fisher Scientific, Waltham, MA, USA).

To synthesize cDNA, the reverse transcription reaction was performed using 1 µg of RNA, oligo dT, dNTP, and recombinant M-MuLV reverse transcriptase (Thermo Fisher Scientific). To analyze microRNA expression, the MystiCq^®^ microRNA cDNA Synthesis Mix (Sigma-Aldrich) was used following the manufacturer’s instructions.

Quantitative real-time PCR (qRT-PCR) was performed with Applied Biosystems^TM^ StepOnePlus Real-Time PCR (Thermo Fisher Scientific) in a 15 µL mixture containing 20 ng of cDNA, SYBR Green PCR Master Mix (Sigma-Aldrich), and forward and reverse primers that recognize murine genes (Table 1), as well as muscle-specific murine microRNAs (Table 2) and nuclease-free water. A universal reverse primer for miRNA qPCR was supplied by a vendor. To verify the purity of the reagents, negative controls containing water instead of cDNA were prepared for each of the genes analyzed. Gene expression levels were calculated by normalizing to the level of housekeeping gene elongation factor 2 (*Eef2*) or constitutive small nuclear RNA U6 (U6 snRNA) in the case of microRNAs. The relative level of gene expression was quantified based on the comparative Ct method (threshold cycle value, defined as the cycle number in which the fluorescence signal crosses the fluorescence threshold) according to the equation: ΔC_T_ = C_T__(gene of interest)_ − C_T__(reference gene)_. In the next step, the relative expression was calculated using the formula 2^−ΔCT^.

### 2.8. Western Blot Analysis

To assess the NRF2 level, 25 µg of protein isolated from C2C12 and C2C12-NRF2 cells were loaded onto a 12% SDS-PAGE gel. After electrophoresis (100 V), dry transfer to the nitrocellulose membrane was performed using the iBlot Dry Blotting System (Invitrogen, Waltham, MA, USA). The membranes were then blocked with 5% milk in Tris-buffered saline (TBS) for 1 h at room temperature (RT). Subsequently, the membranes were incubated overnight at 4 °C with primary antibodies rabbit anti-NRF2 (D1Z9C; Cell Signaling Technology, Danvers, MA, USA; molecular weight ~100 kDa) and mouse anti-α-tubulin (DM1A; Cell Signaling Technology; molecular weight ~52 kDa) diluted 1:100 or 1:1000 in blocking solution, respectively. The next day, the membranes were washed 3 times (5 min each) with TBS + 0.1% Tween 20 and then secondary antibodies conjugated with HRP, goat anti-rabbit (1:1000; Cell Signaling Technology) and goat anti-mouse (1:10,000; BD Pharmingen, San Diego, CA, USA ), were applied for 1 h at RT. The membranes were washed 4 times with TBS and incubated with SuperSignal West Pico Chemiluminescent Substrate (Thermo Fisher Scientific) for 10 min in the dark and finally developed manually on the X-ray film. PageRuler Prestained (Thermo Fisher Scientific) protein ladder was used as a marker.

### 2.9. Immunofluorescent Staining

To analyze the level of C2C12 and mSC differentiation, myosin heavy chain (MHC) and myogenin staining was performed according to our previously applied protocol [22,24,27,32] by overnight incubation at 4 °C with the primary antibodies, rabbit anti-myogenin (1:200, M-225, Santa Cruz, Dallas, TX, USA) and/or mouse anti-MHC (1:200, MY-32, Sigma-Aldrich). The next day, cells were washed 3 times per 5 min with PBS and secondary antibodies, goat anti-rabbit Alexa Fluor 568 (A-11077, Thermo Fisher Scientific) and/or goat anti-mouse Alexa Fluor 488 (A11008, Thermo Fisher Scientific) diluted 1:400 in PBS, were added for 2 h. Subsequently, cells were washed 3 times with PBS with the addition of Hoechst (Sigma-Aldrich) in the second wash and visualized under a fluorescent microscope (Leica DMI6000B). To analyze cell differentiation ability, the fusion index, defined as the percentage of nuclei within myotubes that contain 3 or more nuclei related to the total number of nuclei, was calculated using *ImageJ* software (version 1.53k, Wayne Rasband NIH, Kensington, MD, USA).

### 2.10. Statistical Analysis

Data are presented as mean ± SEM and analyzed with an unpaired two-tailed Student’s *t*-test to determine differences between two groups or a one-way ANOVA followed by Tukey’s post hoc test for multiple groups. All experiments on C2C12 cells were performed in at least duplicates and repeated 2–3 times, while analysis of mSCs was conducted using 4–5 mice/group. The details of the repetition of the experiments are indicated in the figure legends. When comparing two groups with non-normal distribution, the Mann–Whitney test was used. The results were considered statistically significant with a *p*-value ≤ 0.05. Grubb’s test was used to identify significant outliers and GraphPad Prism 8 was applied for graphs and statistical analyzes.

## 3. Results

### 3.1. Effect of NRF2 on the Proliferation, ROS Level and Viability of C2C12 Myoblasts

**Figure 1:**
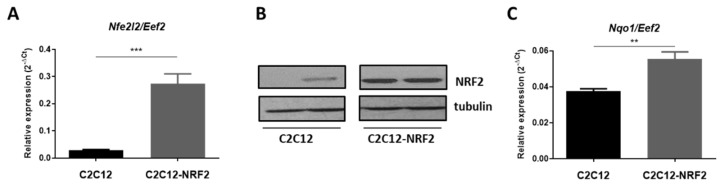
Confirmation of NRF2 (*Nfe2l2*) transgene overexpression. (**A**) mRNA level of *Nfe2l2* gene; qRT-PCR. (**B**) Western blot analysis of NRF2. Tubulin was used as a loading control. (**C**) mRNA level of *Nqo1* gene; qRT-PCR. Data are presented as mean ± SEM; *n* = 2–3; ** *p* ≤ 0.01; *** *p* ≤ 0.005; Student’s *t*-test.

First, we confirmed the overexpression of NRF2 in the modified cell line. Real-time PCR showed an increased expression of *Nfe2l2* in C2C12-NRF2 cells compared to the control cell line (Figure 1A). The protein level was also higher, as shown by the exemplary Western blot results (Figure 1B). Consequently, the expression of the NRF2 target gene, NAD(P)H:quinone oxidoreductase 1 (*Nqo1*), was statistically increased in cells with NRF2 overexpression (Figure 1C).

**Figure 2:**
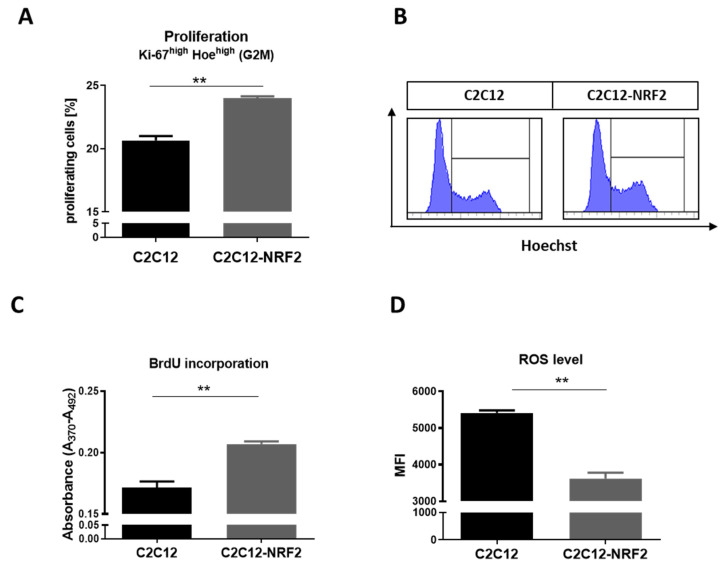
Proliferation and ROS production in C2C12 and C2C12-NRF2 cells. (**A**) Flow cytometry analysis using Ki-67-FITC and Hoechst. (**B**) Representative dot plots of cells with high Hoechst content. (**C**) Proliferation was evaluated by the incorporation of BrdU. (**D**) ROS level was analyzed by the CellROX Flow Cytometry Assay Kit. Data are presented as mean ± SEM; *n* = 2–3; ** *p* ≤ 0.01; Student’s *t*-test.

Then, to assess the effect of NRF2 on myoblast proliferation, C2C12 and C2C12-NRF2 cells were stained with Ki-67-FITC antibody and Hoechst dye and analyzed by flow cytometry. An increased number of cells with a high level of Ki-67 expression and Hoechst binding (cells in the G2 and M phases of the cell cycle) were found in C2C12-NRF2 compared to C2C12 myoblasts (Figure 2A,B). Additionally, BrdU incorporation was higher in cells with NRF2 overexpression (Figure 2C). Furthermore, in these cells, the decreased ROS level was evident by flow cytometry (Figure 2D).

**Figure 3:**
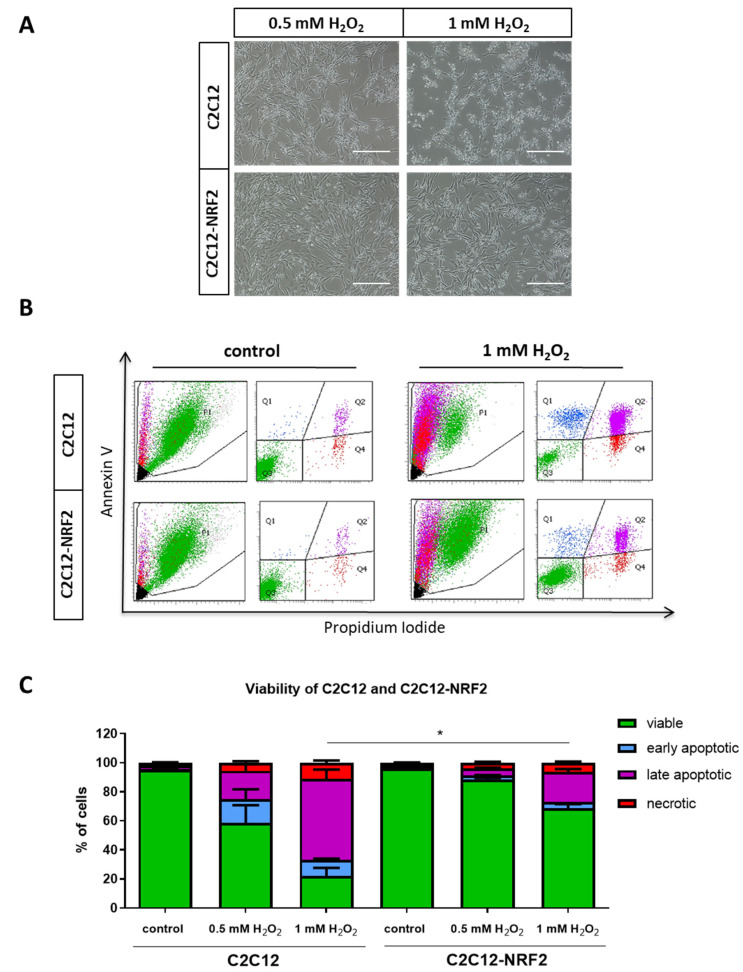
The effect of H_2_O_2_ on mortality of C2C12 and C2C12-NRF2 cell lines. (**A**) Morphology of myoblasts after stimulation with 0.5 mM and 1 mM H_2_O_2_. Contrast phase microscopy. Representative photos. The scale bars represent 100 µm. (**B**) Analysis of live cells (PI^−^Annexin V^−^; green), early apoptotic (PI^−^Annexin V^+^; blue), late apoptotic (PI^+^Annexin V^+^; purple) and necrotic (PI^+^Annexin V^−^; red). Cytometric analysis using propidium iodide (PI) and annexin V, representative dot plots. (**C**) Quantitative analysis (percentage of total cells). Data are presented as mean ± SEM; *n* = 2; * *p* ≤ 0.05 for viable, early apoptotic, late apoptotic, and necrotic cells; Student’s *t*-test.

Finally, cell viability under oxidative stress conditions was examined. Incubation with various concentrations of hydrogen peroxide (0.5–1 mM H_2_O_2_) for 24 h resulted in cell death in both cell lines analyzed but the toxicity was higher in the control cells compared to C2C12-NRF2 myoblasts, which was already visible under the microscope (Figure 3A). Subsequently, cytometric analysis using Annexin V and propidium iodide (PI) staining confirmed the microscopic observation. Analysis of the percentage of viable (PI^−^Annexin V^−^), early apoptotic (PI^−^Annexin V^+^), late apoptotic (PI^+^Annexin V^+^), and necrotic (PI^+^Annexin V^−^) cells revealed a cytoprotective effect of NRF2 overexpression. C2C12-NRF2 cells were significantly more resistant to H_2_O_2_ than control cells (Figure 3B,C).

### 3.2. Effect of NRF2 Overexpression on Differentiation of C2C12 Myoblasts

**Figure 4:**
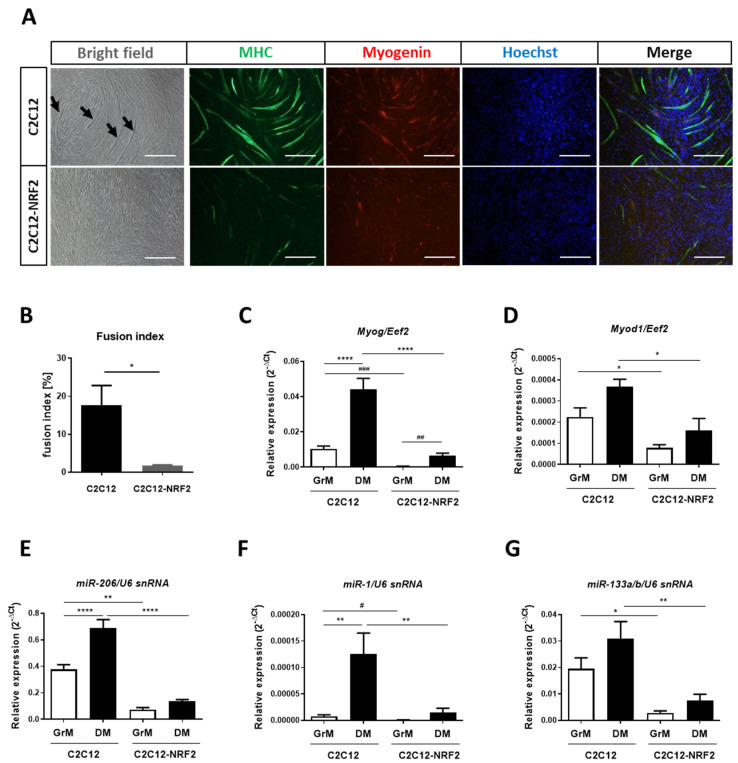
Differentiation of C2C12 and C2C12-NRF2 cell lines. (**A**) Immunofluorescence staining for MHC and myogenin. Hoechst was used to visualize the nuclei. Representative photos. The scale bars represent 100 µm. (**B**) Fusion index of differentiated C2C12 and C2C12-NRF2 cells. Expression of the (**C**) *Myog*, (**D**) *Myod1*, (**E**) miR-206, (**F**) miR-1, and (**G**) miR-133a/b mRNA levels; qRT-PCR. Data are presented as mean ± SEM; *n* = 3; * *p* ≤ 0.05; ** *p* ≤ 0.01; **** *p* ≤ 0.0001; one-way ANOVA with Tukey’s post hoc test. # *p* ≤ 0.05; ## *p* ≤ 0.01; ### *p* ≤ 0.005; Student’s *t*-test. GrM—growth medium, DM—differentiation medium.

In the next step, we checked how NRF2 overexpression affects the differentiation of C2C12 cells. When the cells reached confluence, the medium was changed from growth medium (GrM) to differentiation medium (DM) and the C2C12 and C2C12-NRF2 cell lines were incubated for 5 days and then fixed and analyzed. In control cells, the formation of long mature myotubes (black arrows; contrast phase microscopy) was observed, while in cells overexpressing NRF2, such myotubes were not found (Figure 4A).

These observations were confirmed by immunofluorescence staining for myosin heavy chain (MHC) and myogenin, markers of mature myotubes. The tubes from the C2C12 cell line expressed MHC and myogenin at a higher level compared to the C2C12-NRF2 myoblasts (Figure 4A,B). Subsequently, the levels of *Myog* and *Myod1* mRNA were checked. Expression of both genes was elevated in cells cultured in DM compared to GrM in both cell lines. In cells overexpressing NRF2, the expression of *Myog* (Figure 4C) and *Myod1* (Figure 4D) decreased significantly when cells were cultured both in growth medium and under differentiating conditions. The same pattern was observed for the expression of muscle-specific microRNAs. It should be noted that the expression of miR-206 (Figure 4E), miR-1 (Figure 4F), and miR-133a/b (Figure 4G) was significantly negatively regulated in NRF2-overexpressing cells compared to the control C2C12 cell line.

### 3.3. The Transcriptional Deficiency of NRF2 Affects the Viability of mSC Cultured in Normoxia

**Figure 5:**
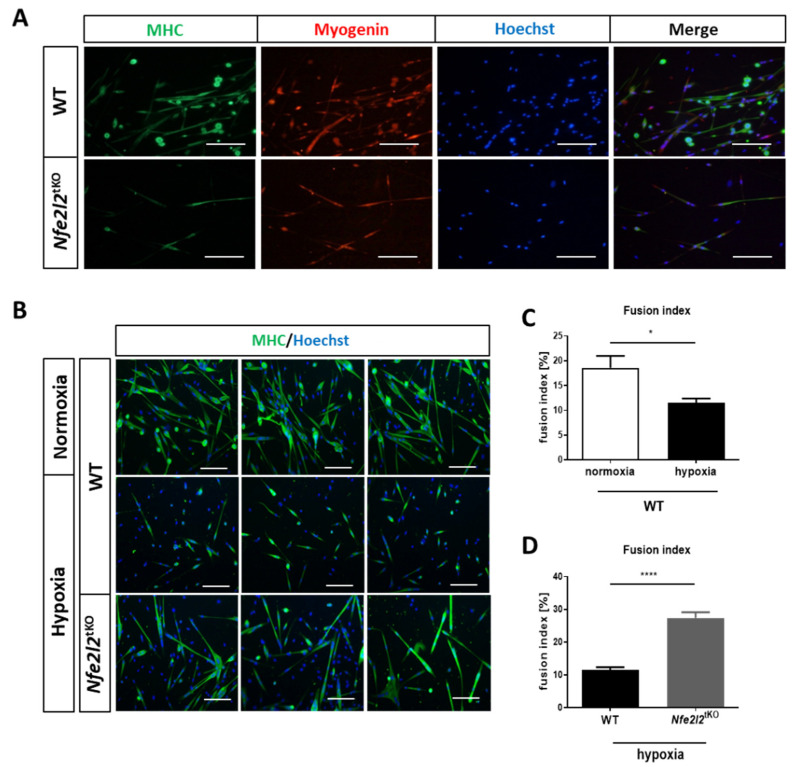
Differentiation of mSCs isolated from hind limb muscles of WT and *Nfe2l2*^tKO^ mice. (**A**) Immunofluorescence staining for MHC and myogenin of cells cultured under normoxic conditions. Hoechst was used to visualize the nuclei. Representative photos. The scale bars represent 100 µm. (**B**) Effect of hypoxic (0.5% O_2_) conditions on the differentiation of mSCs isolated from the hind limb muscles of WT and *Nfe2l2*^tKO^ mice. Immunofluorescence staining for MHC after 3 days of differentiation. Hoechst was used to visualize the nuclei. Representative photos. The scale bars represent 100 µm. (**C**,**D**) Fusion index of differentiated in vitro mSCs. Data are presented as mean ± SEM; *n* = 4–5; * *p* ≤ 0.05; **** *p* ≤ 0.0001; Student’s *t*-test.

In C2C12 immortalized myoblasts, we observed a pro-proliferative and cytoprotective effect of NRF2. To better understand the impact of NRF2 on mSC viability, we utilized the model of NRF2 deficiency. For this purpose, mSCs were isolated from muscles from WT (wild-type) and *Nfe2l2*^tKO^ mice (those without transcriptionally active NRF2 [29]). Primary mSCs isolated from WT animals were viable and differentiated after incubation in DM; however, the growth and viability of mSCs isolated from *Nfe2l2*^tKO^ mice were severely impaired. Only a few cells were found in wells where the mSCs from *Nfe2l2* (transcriptionally)-deficient mice were seeded, indicating that NRF2 is essential for mSC survival and viability (Figure 5A).

### 3.4. The Differentiation of mSCs Isolated from Nfe2l2^tKO^ Mice Is Enhanced under Hypoxic Conditions

Tissue hypoxia is an important factor that regulates stem cell differentiation in the early stages of embryonic development [33]. As the redox status can also affect the culture of primary cells, we checked the effect of a low oxygen concentration on the differentiation of mSCs. In hypoxia (0.5% O_2_), the differentiation of mSCs isolated from WT mice was inhibited, as shown by immunofluorescent staining and quantification of the fusion index (Figure 5B,C). However, mSCs isolated from *Nfe2l2*^tKO^ and then cultured and differentiated under hypoxic conditions were viable and much more differentiated than mSCs from WT animals (Figure 5B). These observations were confirmed by the value of the fusion index, which was significantly higher in the case of *Nfe2l2*^tKO^ than in WT (Figure 5D).

## 4. Discussion

NRF2 transcription factor regulates cellular oxidative balance by orchestrating the expression of many antioxidant and anti-inflammatory genes, including HO-1 [1]. Since we previously showed the strong effect of the latter on the proliferation and differentiation of myoblasts [23], we then aimed to investigate the possible role of its transcriptional regulator in skeletal muscle cells. We found that NRF2 overexpression increases proliferation and viability under oxidative stress, while it inhibits C2C12 myoblast differentiation. Importantly, we also revealed the effect of NRF2 transcriptional deficiency on the functions of primary mSCs. Collectively, our results suggest that this transcription factor may be a possible therapeutic target to protect against disorders related to muscle stem cell dysfunction.

Oxidative stress is one of the major factors that induces apoptosis and inhibits mSC proliferation [34,35,36]. Concomitantly, improved survival of some types of progenitor cells was shown to be achieved by increasing the expression of antioxidant genes [37]. For example, glutathione peroxidase (GPX) protected C2C12 cells [38] and mSCs [39] against apoptosis, while its absence increased their susceptibility to oxidative stress and decreased proliferation [40]. Furthermore, HO-1 improved the survival of transplanted primary myoblasts [41] and its pharmacological inhibition reduced the viability of H_2_O_2_-treated myoblasts [42]. As NRF2 induces the expression of HO-1 [28], the impact of NRF2 on myoblasts, similar to that which we previously showed for HO-1, suggests that the effects exerted by NRF2 depend on HO-1. However, detailed mechanisms require further elucidation. Our observation of an increased proliferation of cells overexpressing NRF2 remains in line with the work by Al-Sawaf et al. [21], which demonstrates enhanced proliferative potential after the transfection of C2C12 cells with shRNA against Keap1, a negative regulator of NRF2. As mSCs reduce the rate of cell division under oxidative stress conditions [40,43,44], it could be assumed that the observed effect is mediated by decreased ROS production in C2C12-NRF2 cells. In fact, we demonstrated suppressed ROS generation in cells overexpressing NRF2 and greater viability of these cells after incubation with H_2_O_2_. Additionally, we found that the formation of mature multinucleated myotubes was potently reduced in cells with a high NRF2 level. Concomitantly, the expression of MRFs, such as *Myod1* and *Myog*, factors involved in the induction of differentiation [45], and myomiRs (miR-1, miR-133a/b, and miR-206) was significantly decreased in myoblasts overexpressing NRF2. We have previously shown similar cytoprotective effects of HO-1, manifested by improved survival of C2C12 cells after oxidative stress and their increased proliferation [23]. The results, which indicate that NRF2 overexpression inhibits C2C12 myoblast differentiation, are also in agreement with the effect of HO-1, as a strong negative influence of HO-1 on myoblast differentiation was evident [23]. Importantly, as we showed, it was not mediated by the antioxidant properties of HO-1, although it still depended on its enzymatic activity. Carbon monoxide, a product of heme degradation, was responsible for the inhibition of the activity of c/EBPδ, the major transcription factor that regulates MYOD expression. Furthermore, HO-1 was found to have a profound effect on miRNAs, negatively regulating their biogenesis and also inhibiting myomiR expression [23]. Therefore, the involvement of a similar non-antioxidant mechanism in cells overexpressing NRF2 cannot be excluded.

In the present work, we evaluated the effect of the genetic overexpression of NRF2. Concomitantly, other studies indicate the protective role of NRF2 induction by the use of active antioxidant substances, such as apple polyphenol, phloretin [46], or platycodin D, a triterpenoid saponin [47] in C2C12 myoblasts. These findings provide insight into the application of NRF2-inducing agents to alleviate oxidative damage in myoblasts and mSCs. However, it should be emphasized that such compounds do not work exclusively through NRF2 activation but could also involve other mechanisms. Interestingly, in the work by Rajasekaran et al. [48] treatment of C2C12 cells with sulforaphane (SFN), a potent NRF2 inducer, not only resulted in the inhibition of cell differentiation but also in strong up-regulation of the reduced glutathione level (GSH) leading to increased reductive stress. Therefore, optimal induction of the antioxidant response and an appropriate intracellular redox environment are necessary for proper myogenic differentiation and efficient regeneration.

An important factor that regulates the activity of mSCs during muscle regeneration could be the hypoxic environment [9]. We show that the differentiation of mSCs cultured in hypoxia (0.5% O_2_) is lower compared to cells grown under normoxic conditions (21% O_2_). On the other hand, the viability of mSCs isolated from mice lacking transcriptionally active NRF2 was strongly altered in atmospheric oxygen concentration, while it improved greatly in hypoxia. It has been shown that maintaining the quiescent and undifferentiated state of some types of primary cells is facilitated under hypoxic conditions [49]. However, there are many discrepancies in the literature regarding the effect of hypoxia on myoblast proliferation and differentiation. One of the first studies report that low oxygen tension improves the proliferation of murine [50] and rat [51] muscle precursor cells. However, it was not confirmed in the case of C2C12 myoblasts [52] and human mSCs [53]. Both studies showed a decrease in the proliferation and differentiation of the tested cells. These differences suggested that the effect of oxygen pressure on proliferation and differentiation could be cell- and species-dependent. Furthermore, oxygen level has been shown to increase mSC quiescence and self-renewal, while hypoxia-conditioned myoblasts have better transplantation efficiency [54].

We found that mSCs isolated from mice lacking NRF2 transcriptional activity and cultured under hypoxic conditions are viable and more differentiated than cells isolated from WT mice. Several mechanisms responsible for changes in muscle regeneration and mSC functions due to hypoxia—including differences in MRF expression [52,55] and the involvement of hypoxia-inducible factor (HIF)-1 and HIF-2, transcriptional factors that mediate the cellular response to hypoxia [56]—have already been proposed. One of the possible regulations may involve mitophagy, the mitochondria-directed autophagy. Zhang et al. demonstrated that hypoxia-induced extensive mitochondrial degradation protects the heart from the consequences of ischemia-reperfusion injury [57]. The balance between mitophagy and mitochondrial biogenesis in the hypoxic stress response and control of the ROS level may be crucial for cell differentiation. Although we demonstrated that the lack of *Hmox1* does not affect autophagy and mitophagy markers in dystrophic muscles [58], it does not exclude the possible role of NRF2 in hypoxia-regulated mSC differentiation. It could also be hypothesized that the observed changes in mSC differentiation may result from hypoxia-induced metabolic alterations. In hypoxia, cells preferentially use the O_2_-independent glycolytic pathway to maintain sufficient ATP production. This metabolic adaptation may influence various aspects of the fate of mSCs (quiescence, activation, proliferation, migration, differentiation, fusion, and self-renewal). Furthermore, lactate, one of the metabolic products of glycolysis, may also regulate myogenic differentiation through different mechanisms (reviewed in [59]). The NRF2 pathway is known to play a role in activating and maintaining the HIF-1 response and we have previously demonstrated crosstalk between these factors in endothelial cells [60]. NRF2 can change oxidative phosphorylation to glycolytic energy production through HIF-1α induction and regulate the expression of genes that encode enzymes involved in glycolysis and other metabolic pathways. Therefore, more studies are needed to evaluate the possibility of metabolic-dependent regulation of cell differentiation. Interestingly, recent findings suggest that HIF-2α is also a crucial mediator of hypoxia signaling in mSCs [61]. Therefore, our current work adds to the suggestion that the link between HIF-1 and/or HIF-2 and NRF2 could also be crucial for myoblast functions.

## 5. Conclusions

In summary, we showed that the NRF2 transcription factor affects the properties of murine myoblasts and mSCs. Our studies revealed that NRF2 overexpression in C2C12 myoblasts decreases ROS production, as well as improves proliferation and viability under oxidative stress. We found that NRF2 is essential for the viability of mSCs cultured under normoxic conditions, while under hypoxia, the lack of transcriptionally active NRF2 increases the differentiation of primary cultured mSCs. This indicates that direct modulation of NRF2, through genetic and/or pharmacological overexpression, can influence the redox state and functions of mSCs.

## Figures and Tables

**Table 1:** Sequences of primers used for qPCR analysis.

Gene	Forward Primer 5′–3′	Reverse Primer 3′–5′
*Eef2*	AGAACATATTATTGCTGGCG	CAACAGGGTCAGATTTCTTG
*Myod1*	GCTGCCTTCTACGCACCTG	GCCGCTGTAATCCATCATGC
*Myog*	CAGTACATTGAGCGCCTACAG	GGACCGAACTCCAGTGCAT
*Nfe2l2*	TCACACGAGATGAGCTTAGGGCAA	TACAGTTCTGGGCGGCGACTTTAT
*Nqo1*	AGGACCCTTCCGGAGTAAGA	CCAGGATTTGAAATTCGGGGCG

**Table 2:** Sequences of primers used for miRNA-specific qPCR analysis.

miRNA Name	Forward Primer 5′–3′
miR-1	GCTGGAATGTAAAGAAGTATGTAT
miR-133a/b	TGGTCCCCTTCAACCAGCTGT
miR-206	TGGAATGTAAGGAAGTGTGTGG
U6 snRNA	CGCAAGGATGACACGCAAATTC

## Data Availability

Not applicable.

## Supplementary Materials

The following supporting information can be downloaded at: https://www.mdpi.com/article/10.3390/cells11203321/s1, Figure S1: (A) Gating strategy used to sort mSCs from murine skeletal muscle. (B) Morphology of mSC on the first day after sorting and on the third day of differentiation. DM—differentiation medium.

## References

[1] Loboda A., Damulewicz M., Pyza E., Jozkowicz A., Dulak J. (2016). Role of Nrf2/HO-1 System in Development, Oxidative Stress Response and Diseases: An Evolutionarily Conserved Mechanism. Cell. Mol. Life Sci..

[2] Cuadrado A., Manda G., Hassan A., Alcaraz M.J., Barbas C., Daiber A., Ghezzi P., León R., López M.G., Oliva B. (2018). Transcription Factor NRF2 as a Therapeutic Target for Chronic Diseases: A Systems Medicine Approach. Pharmacol. Rev..

[3] Hayes J.D., Dinkova-Kostova A.T. (2014). The Nrf2 Regulatory Network Provides an Interface between Redox and Intermediary Metabolism. Trends Biochem. Sci..

[4] Łoboda A., Dulak J. (2020). Muscle and Cardiac Therapeutic Strategies for Duchenne Muscular Dystrophy: Past, Present, and Future. Pharmacol. Rep..

[5] Petrillo S., Pelosi L., Piemonte F., Travaglini L., Forcina L., Catteruccia M., Petrini S., Verardo M., D’Amico A., Musarò A. (2017). Oxidative Stress in Duchenne Muscular Dystrophy: Focus on the NRF2 Redox Pathway. Hum. Mol. Genet..

[6] Hill J.A., Olson E.N. (2012). Muscle. Fundamental Biology and Mechanisms of the Disease.

[7] Schultz E., Gibson M.C., Champion T. (1978). Satellite Cells Are Mitotically Quiescent in Mature Mouse Muscle: An EM and Radioautographic Study. J. Exp. Zool..

[8] Reznik M. (1969). Thymidine-3H Uptake by Satellite Cells of Regenerating Skeletal Muscle. J. Cell Biol..

[9] Chaillou T., Lanner J.T. (2016). Regulation of Myogenesis and Skeletal Muscle Regeneration: Effects of Oxygen Levels on Satellite Cell Activity. FASEB J..

[10] Yin H., Price F., Rudnicki M.A. (2013). Satellite Cells and the Muscle Stem Cell Niche. Physiol. Rev..

[11] Schiaffino S., Rossi A.C., Smerdu V., Leinwand L.A., Reggiani C. (2015). Developmental Myosins: Expression Patterns and Functional Significance. Skelet. Muscle.

[12] Hernández-Hernández J.M., García-González E.G., Brun C.E., Rudnicki M.A. (2017). The Myogenic Regulatory Factors, Determinants of Muscle Development, Cell Identity and Regeneration. Semin. Cell Dev. Biol..

[13] Nakajima N., Takahashi T., Kitamura R., Isodono K., Asada S., Ueyama T., Matsubara H., Oh H. (2006). MicroRNA-1 Facilitates Skeletal Myogenic Differentiation without Affecting Osteoblastic and Adipogenic Differentiation. Biochem. Biophys. Res. Commun..

[14] Anderson C., Catoe H., Werner R. (2006). MIR-206 Regulates Connexin43 Expression during Skeletal Muscle Development. Nucleic Acids Res..

[15] Chen J.-F., Mandel E.M., Thomson J.M., Wu Q., Callis T.E., Hammond S.M., Conlon F.L., Wang D.-Z. (2006). The Role of MicroRNA-1 and MicroRNA-133 in Skeletal Muscle Proliferation and Differentiation. Nat. Genet..

[16] Yuasa K., Hagiwara Y., Ando M., Nakamura A., Takeda S., Hijikata T. (2008). MicroRNA-206 Is Highly Expressed in Newly Formed Muscle Fibers: Implications Regarding Potential for Muscle Regeneration and Maturation in Muscular Dystrophy. Cell Struct. Funct..

[17] Yamaguchi M., Murakami S., Yoneda T., Nakamura M., Zhang L., Uezumi A., Fukuda S., Kokubo H., Tsujikawa K., Fukada S. (2015). Evidence of Notch-Hesr-Nrf2 Axis in Muscle Stem Cells, but Absence of Nrf2 Has No Effect on Their Quiescent and Undifferentiated State. PLoS ONE.

[18] Miller C.J., Gounder S.S., Kannan S., Goutam K., Muthusamy V.R., Firpo M.A., Symons J.D., Paine R., Hoidal J.R., Rajasekaran N.S. (2012). Disruption of Nrf2/ARE Signaling Impairs Antioxidant Mechanisms and Promotes Cell Degradation Pathways in Aged Skeletal Muscle. Biochim. Biophys. Acta (BBA) Mol. Basis Dis..

[19] Narasimhan M., Hong J., Atieno N., Muthusamy V.R., Davidson C.J., Abu-Rmaileh N., Richardson R.S., Gomes A.V., Hoidal J.R., Rajasekaran N.S. (2014). Nrf2 Deficiency Promotes Apoptosis and Impairs PAX7/MyoD Expression in Aging Skeletal Muscle Cells. Free. Radic. Biol. Med..

[20] Shelar S.B., Narasimhan M., Shanmugam G., Litovsky S.H., Gounder S.S., Karan G., Arulvasu C., Kensler T.W., Hoidal J.R., Darley-Usmar V.M. (2016). Disruption of Nuclear Factor (Erythroid-Derived-2)-like 2 Antioxidant Signaling: A Mechanism for Impaired Activation of Stem Cells and Delayed Regeneration of Skeletal Muscle. FASEB J..

[21] Al-Sawaf O., Fragoulis A., Rosen C., Keimes N., Liehn E.A., Hölzle F., Kan Y.W., Pufe T., Sönmez T.T., Wruck C.J. (2014). Nrf2 Augments Skeletal Muscle Regeneration after Ischaemia-Reperfusion Injury: Nrf2 Augments Skeletal Muscle Regeneration after Ischaemia-Reperfusion Injury. J. Pathol..

[22] Bronisz-Budzyńska I., Kozakowska M., Podkalicka P., Kachamakova-Trojanowska N., Łoboda A., Dulak J. (2020). The Role of Nrf2 in Acute and Chronic Muscle Injury. Skelet. Muscle.

[23] Kozakowska M., Ciesla M., Stefanska A., Skrzypek K., Was H., Jazwa A., Grochot-Przeczek A., Kotlinowski J., Szymula A., Bartelik A. (2012). Heme Oxygenase-1 Inhibits Myoblast Differentiation by Targeting Myomirs. Antioxid. Redox Signal..

[24] Pietraszek-Gremplewicz K., Kozakowska M., Bronisz-Budzynska I., Ciesla M., Mucha O., Podkalicka P., Madej M., Glowniak U., Szade K., Stępniewski J. (2018). Heme Oxygenase-1 Influences Satellite Cells and Progression of Duchenne Muscular Dystrophy in Mice. Antioxid. Redox Signal..

[25] Takemoto Y., Inaba S., Zhang L., Tsujikawa K., Uezumi A., Fukada S.-I. (2019). Implication of Basal Lamina Dependency in Survival of Nrf2-Null Muscle Stem Cells via an Antioxidative-Independent Mechanism. J. Cell. Physiol..

[26] Igarashi K., Itoh K., Motohashi H., Hayashi N., Matuzaki Y., Nakauchi H., Nishizawa M., Yamamoto M. (1995). Activity and Expression of Murine Small Maf Family Protein MafK. J. Biol. Chem..

[27] Kozakowska M., Pietraszek-Gremplewicz K., Ciesla M., Seczynska M., Bronisz-Budzynska I., Podkalicka P., Bukowska-Strakova K., Loboda A., Jozkowicz A., Dulak J. (2018). Lack of Heme Oxygenase-1 Induces Inflammatory Reaction and Proliferation of Muscle Satellite Cells after Cardiotoxin-Induced Skeletal Muscle Injury. Am. J. Pathol..

[28] Itoh K., Chiba T., Takahashi S., Ishii T., Igarashi K., Katoh Y., Oyake T., Hayashi N., Satoh K., Hatayama I. (1997). An Nrf2/Small Maf Heterodimer Mediates the Induction of Phase II Detoxifying Enzyme Genes through Antioxidant Response Elements. Biochem. Biophys. Res. Commun..

[29] Kloska D., Kopacz A., Cysewski D., Aepfelbacher M., Dulak J., Jozkowicz A., Grochot-Przeczek A. (2019). Nrf2 Sequesters Keap1 Preventing Podosome Disassembly: A Quintessential Duet Moonlights in Endothelium. Antioxid. Redox Signal..

[30] Innamorato N.G., Jazwa A., Rojo A.I., García C., Fernández-Ruiz J., Grochot-Przeczek A., Stachurska A., Jozkowicz A., Dulak J., Cuadrado A. (2010). Different Susceptibility to the Parkinson’s Toxin MPTP in Mice Lacking the Redox Master Regulator Nrf2 or Its Target Gene Heme Oxygenase-1. PLoS ONE.

[31] Chomczynski P., Sacchi N. (1987). Single-Step Method of RNA Isolation by Acid Guanidinium Thiocyanate-Phenol-Chloroform Extraction. Anal. Biochem..

[32] Podkalicka P., Mucha O., Bronisz-Budzyńska I., Kozakowska M., Pietraszek-Gremplewicz K., Cetnarowska A., Głowniak-Kwitek U., Bukowska-Strakova K., Cieśla M., Kulecka M. (2020). Lack of MiR-378 Attenuates Muscular Dystrophy in Mdx Mice. JCI Insight.

[33] Podkalicka P., Stępniewski J., Mucha O., Kachamakova-Trojanowska N., Dulak J., Łoboda A. (2020). Hypoxia as a Driving Force of Pluripotent Stem Cell Reprogramming and Differentiation to Endothelial Cells. Biomolecules.

[34] Ardite E., Barbera J.A., Roca J., Fernández-Checa J.C. (2004). Glutathione Depletion Impairs Myogenic Differentiation of Murine Skeletal Muscle C2C12 Cells through Sustained NF-KappaB Activation. Am. J. Pathol..

[35] Mofarrahi M., Brandes R.P., Gorlach A., Hanze J., Terada L.S., Quinn M.T., Mayaki D., Petrof B., Hussain S.N.A. (2008). Regulation of Proliferation of Skeletal Muscle Precursor Cells by NADPH Oxidase. Antioxid. Redox Signal..

[36] Urish K.L., Vella J.B., Okada M., Deasy B.M., Tobita K., Keller B.B., Cao B., Piganelli J.D., Huard J. (2009). Antioxidant Levels Represent a Major Determinant in the Regenerative Capacity of Muscle Stem Cells. Mol. Biol. Cell.

[37] Yao E.-H., Yu Y., Fukuda N. (2006). Oxidative Stress on Progenitor and Stem Cells in Cardiovascular Diseases. Curr. Pharm. Biotechnol..

[38] Nishida H., Ichikawa H., Konishi T. (2007). Shengmai-San Enhances Antioxidant Potential in C2C12 Myoblasts through the Induction of Intracellular Glutathione Peroxidase. J. Pharmacol. Sci..

[39] Pallafacchina G., François S., Regnault B., Czarny B., Dive V., Cumano A., Montarras D., Buckingham M. (2010). An Adult Tissue-Specific Stem Cell in Its Niche: A Gene Profiling Analysis of in Vivo Quiescent and Activated Muscle Satellite Cells. Stem Cell Res..

[40] Lee S., Shin H.S., Shireman P.K., Vasilaki A., Van Remmen H., Csete M.E. (2006). Glutathione-Peroxidase-1 Null Muscle Progenitor Cells Are Globally Defective. Free Radic. Biol. Med..

[41] Laumonier T., Yang S., Konig S., Chauveau C., Anegon I., Hoffmeyer P., Menetrey J. (2008). Lentivirus Mediated HO-1 Gene Transfer Enhances Myogenic Precursor Cell Survival after Autologous Transplantation in Pig. Mol. Ther..

[42] Aggeli I.-K., Kefaloyianni E., Beis I., Gaitanaki C. (2010). HOX-1 and COX-2: Two Differentially Regulated Key Mediators of Skeletal Myoblast Tolerance under Oxidative Stress. Free Radic. Res..

[43] Scimè A., Desrosiers J., Trensz F., Palidwor G.A., Caron A.Z., Andrade-Navarro M.A., Grenier G. (2010). Transcriptional Profiling of Skeletal Muscle Reveals Factors That Are Necessary to Maintain Satellite Cell Integrity during Ageing. Mech. Ageing Dev..

[44] Hansen J.M., Klass M., Harris C., Csete M. (2007). A Reducing Redox Environment Promotes C2C12 Myogenesis: Implications for Regeneration in Aged Muscle. Cell Biol. Int..

[45] Le Grand F., Rudnicki M.A. (2007). Skeletal Muscle Satellite Cells and Adult Myogenesis. Curr. Opin. Cell Biol..

[46] Li J., Yang Q., Han L., Pan C., Lei C., Chen H., Lan X. (2020). C2C12 Mouse Myoblasts Damage Induced by Oxidative Stress Is Alleviated by the Antioxidant Capacity of the Active Substance Phloretin. Front. Cell Dev. Biol..

[47] Choi Y.H. (2020). Activation of the Nrf2/HO-1 Signaling Pathway Contributes to the Protective Effects of Platycodin D against Oxidative Stress-Induced DNA Damage and Apoptosis in C2C12 Myoblasts. Gen. Physiol. Biophys..

[48] Rajasekaran N.S., Shelar S.B., Jones D.P., Hoidal J.R. (2020). Reductive Stress Impairs Myogenic Differentiation. Redox Biol..

[49] Mohyeldin A., Garzón-Muvdi T., Quiñones-Hinojosa A. (2010). Oxygen in Stem Cell Biology: A Critical Component of the Stem Cell Niche. Cell Stem Cell.

[50] Chakravarthy M.V., Spangenburg E.E., Booth F.W. (2001). Culture in Low Levels of Oxygen Enhances in Vitro Proliferation Potential of Satellite Cells from Old Skeletal Muscles. Cell. Mol. Life Sci..

[51] Lees S.J., Childs T.E., Booth F.W. (2008). P21^Cip1^ Expression Is Increased in Ambient Oxygen, Compared to Estimated Physiological (5%) Levels in Rat Muscle Precursor Cell Culture. Cell Prolif..

[52] Di Carlo A., De Mori R., Martelli F., Pompilio G., Capogrossi M.C., Germani A. (2004). Hypoxia Inhibits Myogenic Differentiation through Accelerated MyoD Degradation. J. Biol. Chem..

[53] Launay T., Hagström L., Lottin-Divoux S., Marchant D., Quidu P., Favret F., Duvallet A., Darribère T., Richalet J.P., Beaudry M. (2010). Blunting Effect of Hypoxia on the Proliferation and Differentiation of Human Primary and Rat L6 Myoblasts Is Not Counteracted by Epo. Cell Prolif..

[54] Liu W., Wen Y., Bi P., Lai X., Liu X.S., Liu X., Kuang S. (2012). Hypoxia Promotes Satellite Cell Self-Renewal and Enhances the Efficiency of Myoblast Transplantation. Development.

[55] Yun Z., Lin Q., Giaccia A.J. (2005). Adaptive Myogenesis under Hypoxia. Mol. Cell. Biol..

[56] Loboda A., Jozkowicz A., Dulak J. (2010). HIF-1 and HIF-2 Transcription Factors—Similar but Not Identical. Mol. Cells.

[57] Zhang W., Ren H., Xu C., Zhu C., Wu H., Liu D., Wang J., Liu L., Li W., Ma Q. (2016). Hypoxic Mitophagy Regulates Mitochondrial Quality and Platelet Activation and Determines Severity of I/R Heart Injury. Elife.

[58] Mucha O., Kaziród K., Podkalicka P., Rusin K., Dulak J., Łoboda A. (2021). Dysregulated Autophagy and Mitophagy in a Mouse Model of Duchenne Muscular Dystrophy Remain Unchanged Following Heme Oxygenase-1 Knockout. Int. J. Mol. Sci..

[59] Nalbandian M., Radak Z., Takeda M. (2020). Lactate Metabolism and Satellite Cell Fate. Front. Physiol..

[60] Loboda A., Stachurska A., Florczyk U., Rudnicka D., Jazwa A., Wegrzyn J., Kozakowska M., Stalinska K., Poellinger L., Levonen A.-L. (2009). HIF-1 Induction Attenuates Nrf2-Dependent IL-8 Expression in Human Endothelial Cells. Antioxid. Redox Signal..

[61] Xie L., Yin A., Nichenko A.S., Beedle A.M., Call J.A., Yin H. (2018). Transient HIF2A Inhibition Promotes Satellite Cell Proliferation and Muscle Regeneration. J. Clin. Investig..

