# Supplementary material for: NRF2 Regulates Viability, Proliferation, Resistance to Oxidative Stress, and Differentiation of Murine Myoblasts and Muscle Satellite Cells
Source: Cells. 2022 Oct 21;11(20):3321. doi: 10.3390/cells11203321 (PMC9600498; doi:10.3390/cells11203321)
Supplement: Supplementary file 1 [file cells-11-03321-s001.zip › cells-1951548-supplementary.pdf]

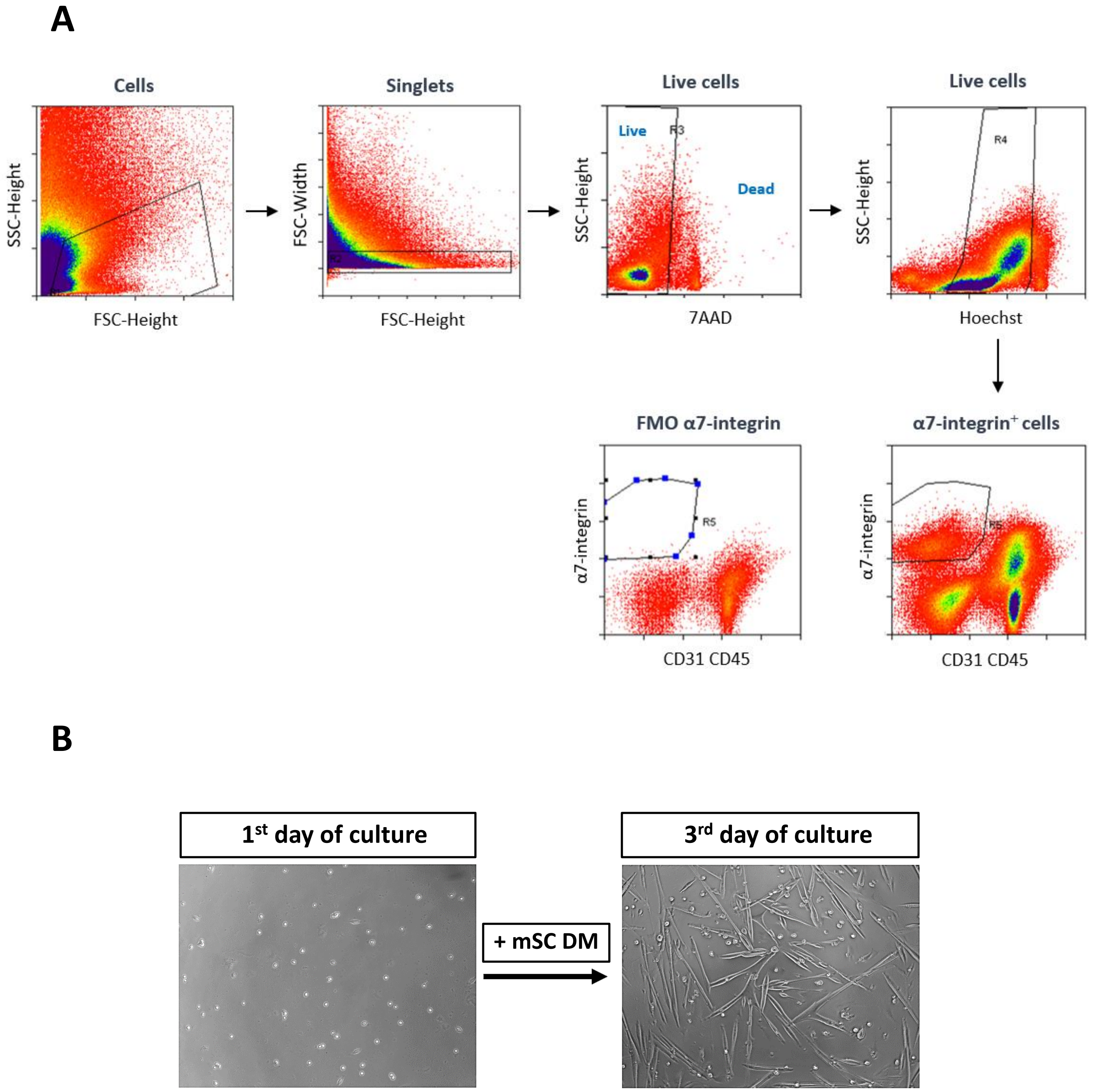

Figure S1: (A) Gating strategy used to sort mSCs from murine skeletal muscle. (B) Morphology of mSC on the first day after sorting and on the third day of differentiation. DM—differentiation medium.
